# Gene expression analyses of the small intestine of pigs in the ex-evacuation zone of the Fukushima Daiichi Nuclear Power Plant

**DOI:** 10.1186/s12917-017-1263-5

**Published:** 2017-11-15

**Authors:** Motoko Morimoto, Ayaka Kato, Jin Kobayashi, Kei Okuda, Yoshikazu Kuwahara, Yasushi Kino, Yasuyuki Abe, Tsutomu Sekine, Tomokazu Fukuda, Emiko Isogai, Manabu Fukumoto

**Affiliations:** 1 School of Food, Agricultural, and Environmental Sciences, Miyagi University, 2-2-1 Hatatate, Taihaku-Ku, Sendai, Miyagi 982-0215 Japan; 2 Institute of Environmental Radioactivity, Fukushima University, Kanayagawa, Fukushima, Japan; 3 Department of Pathology, Institute of Development, Aging and Cancer, Tohoku University, Sendai, Miyagi Japan; 4 Department of Chemistry, Tohoku University, Sendai, Miyagi Japan; 5 Faculty of Life and Environmental Sciences, Prefectural University of Hiroshima, Shobara, Hiroshima Japan; 6 Institute for Excellence in Higher Education, Tohoku University, Sendai, Miyagi Japan; 7 Graduate School of Science and Engineering, Iwate University, Morioka, Iwate Japan; 8 Graduate School of Agricultural Sciences, Tohoku University, Sendai, Miyagi Japan

**Keywords:** Radioactive contaminants, Radiation exposure, Evacuation zone, Small intestine

## Abstract

**Background:**

After the accident at the Fukushima Daiichi Nuclear Power Plant, radioactive contaminants were released over a widespread area. Monitoring the biological effects of radiation exposure in animals in the ex-evacuation zone should be continued to understand the health effects of radiation exposure in humans. The present study aimed to clarify the effects of radiation by investigating whether there is any alteration in the morphology and gene expressions of immune molecules in the intestine of pigs and inobuta (wild boar and domestic pig hybrid) in the ex-evacuation zone in 2012. Gene expression analysis was performed in small intestine samples from pigs, which were collected from January to February 2012, in the ex-evacuation zone. Pigs lived freely in this zone, and their small intestine was considered to be affected by the dietary intake of radioactive contaminants.

**Results:**

Several genes were selected by microarray analysis for further investigation using real-time polymerase chain reaction. *IFN-γ*, which is an important inflammatory cytokine, and *TLR3*, which is a pattern recognize receptor for innate immune system genes, were highly elevated in these pigs. The expressions of the genes of these proteins were associated with the radiation level in the muscles. We also examined the alteration of gene expressions in wild boars 5 years after the disaster. The expression of *IFN-γ* and *TLR3* remained high, and that of *Cyclin G1*, which is important in the cell cycle, was elevated.

**Conclusions:**

We demonstrated that some changes in gene expression occurred in the small intestine of animals in the ex-evacuation zone after radiation. It is difficult to conclude that these alterations are caused by only artificial radionuclides from the Fukushima Daiichi Nuclear Power Plant. However, the animals in the ex-evacuation zone might have experienced some changes owing to radioactive materials, including contaminated soil, small animals, and insects. We need to continue monitoring the effects of long-term radiation exposure in living things.

## Background

After the Great East Japan Earthquake on March 11, 2011, a huge amount of radioactive cesium was released following an accident at Fukushima Daiichi Nuclear Power Plant (FNPP) [1], and the Japanese government set the evacuation zone within 20-km radius from FNPP (ex-evacuation zone now). People were seriously concerned about food safety. Therefore, the Ministry of Agriculture, Forestry and Fisheries officially informed the local government around the FNPP about management guidelines for feeds. However, rice hay was contaminated over a large area owing to wind, and many cows were found to be contaminated, including those that were far from FNPP [2]. Consumers required thorough radioactivity inspection for food products. Fukuda et al. previously reported a correlation between ^137^Cs radioactivity in whole peripheral blood and that in the organs of cattle and established an approach to estimate muscle contamination with a small amount of blood [3]. Therefore, the level of contamination in the muscles of cows could be estimated using a blood test. However, many other livestock were abandoned in the evacuation zone at that time. Pigs have a different feeding habit and different physiological properties compared with those of cows. Therefore, raising concern about contaminated rice hay with regard to pigs was not necessary. Pigs are omnivorous animals, and they eat small insects or tree nuts, such as acorn, with soil. Large physiological differences exist between cows and pigs, and it is important to understand the biological effects of radioactive cesium in pigs in the ex- evacuation zone.

The immune system and physiological functions of pigs are very similar to those of humans [4–6]. Therefore, knowledge about the responses in pigs to radioactive contamination can be useful to understand radiation effects and responses in humans. The intestine can be greatly affected by radiation through internal exposure after oral intake of contaminated food. The intestinal epithelium constitutively and greatly proliferates from stem cells, which exist in the crypt [7]. It is well known that proliferative cells are highly sensitive to radiation, and a previous report mentioned that a dose of radiation as low as 0.01–0.05 Gy can induce apoptosis in these cells [7]. The gastrointestinal tract is not only an organ for digestion and absorption of nutrients, but also an important organ in the host defense mechanism. In the gastrointestinal tract, immune and nonimmune cells work together to expel pathogens, as part of the mucosal barrier. In addition, the immune system in the intestine is closely linked to physiological functions, including metabolism and chronic inflammation.

In this study, we focused on the intestine and aimed to clarify the effects of radiation by investigating whether there is any alteration in morphology and gene expressions of immune molecules in the intestine of pigs and inobuta (wild boar and domestic pig hybrid) in the ex-evacuation zone in 2012. Additionally, we assessed samples obtained from wild boars in the ex-evacuation zone in 2015.

## Methods

### Samples

We collected intestine and muscle samples from euthanized 13 pigs between 18 January and 16 February 2012 at 5 km southwest of the FNPP and three inobuta on 28 February 2012 at 8 km southwest of the FNPP. The animals were sacrificed by the following method according to the Regulation for Animal Experiments and Related Activities at Tohoku University by the veterinary doctors belonging to the Livestock Hygiene Service Center of Fukushima Prefecture [2]. Estimates of the amounts of ^134^Cs and ^137^Cs deposited on the ground have been reported [8]. Intestine samples were collected from five wild boars on 18 November 2015, in Namie town. Control intestine samples were obtained from three healthy pigs present in an uncontaminated pigsty in Miyagi Prefecture in 2012. Each experimental protocol was approved by the Institutional Ethics Commissions for Animal Research at Tohoku University, Fukushima University, and Miyagi University.

### Measurement of radioactivity

Radioactivity in the muscle samples was determined with gamma-ray spectrometry using high-purity germanium (HPGe) detectors (Ortec Co., Oak Ridge, TN, USA), as described in our previous report [2]. Gamma rays from ^134^Cs and ^137^Cs were observed and radioactivity ratios of ^134^Cs to ^137^Cs (decay corrected to March 11, 2011) were 0.9–1.0 which corresponded to other samples polluted by the FNPP accident.

### Pathological analysis

Small pieces of the small intestine were slit longitudinally, laid flat with the mucosal surface facing down, and rolled around a wooden stick (Swiss roll). Paraffin blocks were prepared for pathomorphological examination using hematoxylin and eosin (HE) staining and Masson trichrome (MT) staining.

### Gene expression analysis

Total RNA was extracted from whole tissue using TRIzol Reagent (Life Technologies, Inc., Frederic, MD, USA) according to the manufacturer’s instructions. RNA concentration was measured on a NanoDrop spectrophotometer (Thermo Scientific, Wilmington, DE, USA), and cDNA was synthesized with random primers and SuperScript II (Life Technologies, Inc.). cDNA samples were analyzed using microarray methods (V1: 4 × 44 K, Agilent Technologies, Palo Alto, CA, USA), and genes that showed some expression differences between control pigs and those in the ex-evacuation zone were selected for further PCR analysis. Primer sequences were designed using Primer-BLAST with sequences obtained from GenBank, and these are listed in Table 1.

**Table 1:** List of Primers

Gene		Primer sequences (5′ to 3′)
IFN-**γ**	sense antisense	GGCCATTCAAAGGAGCATGG AGTTCACTGATGGCTTTGCG
TLR3	sense antisense	TCACCCTGCCTAGCATTTGA ACAAGGCAAACTCCTGCTCA
Cyclin G1	sense antisense	CGATTCTCCTCGCCTCGTAG CAGGGCATTCAGCTGGTGTA
AIFM1	sense antisense	AGAGTAGCGGTTGCCGAAAT CATGCCATCGCTGGAACAAG
EPHX2	sense antisense	AGTCATCTGCTCCTCCCGAA GCCCTCACTCTCTCAGGGTA
GADD45A	sense antisense	ATGCCCTCGAAGAAGTGCTC CGCTTGGATCAGGGTGAAGT
OGG1	sense antisense	CGTTCTGCCCCGTAGCAT TCCTCAGTCTGGGTCAGTGT
Smad7	sense antisense	AGAGTGGGGAGGCTCTACTG TCTGCACCAGCTGACTCTTG
XAB2	sense antisense	GTGCCGAGGAGTACATCGAG TCCACGTTGAGAGACTGCAC
XPC	sense antisense	GGAAGAAGACGCACCTTCCA TGTGTTCTCTCCCAAGCCAC
XRCC1	sense antisense	GACCCTCCTGCGTCTTTCTG CCAGCTGGAGAACCACAGAG

Real-time PCR was performed using Brilliant SYBR Green QPCR Master Mix III (Stratagene, La Jolla, CA, USA) with an MX3000P system (Stratagene, La Jolla, CA, USA). Amplification conditions were as follows: 95 °C for 3 min, 40–50 cycles at 95 °C for 5 s, and 60 °C for 20 s. Fluorescence signals measured during the amplification were analyzed. Ribosomal RNA primers were used as an internal control, and all data were normalized to constitutive rRNA values. Quantitative differences between the groups were calculated according to the manufacturer’s instructions (Applied Biosystems, Foster City, CA, USA).

### Statistical analysis

All data are presented as mean ± standard error (SE) for each treatment group. Differences in mRNA expression among the groups were determined using the *t*-test (Prism: GraphPad Software Inc., LaJolla, CA, USA). Differences were considered statistically significant at a *P*-value <0.05.

## Results

### Gene expression in the small intestine of pigs in the ex-evacuation zone

**Fig. 1:**
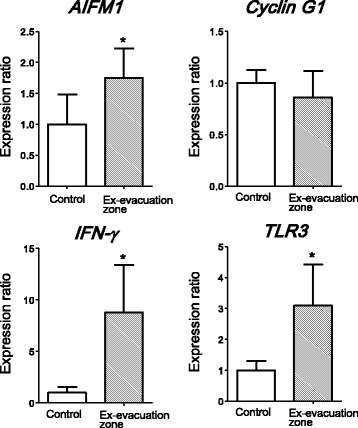
Real-time polymerase chain reaction analysis of *AIFM1*, *Cyclin G1*, *IFN-γ*, and *TLR3* gene expressions in the small intestine of control pigs (*N* = 3) and pigs from the ex-evacuation zone in 2012 (*N* = 13). All data are expressed in relative units compared with control pigs. *P < 0.05. Data are presented as mean ± SE

To identify the genes that were up or down regulated by radiation in the ex-evacuation zone, RNA was extracted for microarray analysis from the small intestine, which is highly sensitive to radiation. Hybridization was performed using pig cDNA 4 × 44 K oligonucleotide microarrays with RNA samples that were collected in 2012. The number of differentially expressed genes with a ≥ 2-fold change was 5135. We selected some genes that were reported as biomarkers for radiation exposure [9, 10], as well as some genes to show the significant expression differences in microarray analysis after radiation, for subsequent analysis using real-time PCR. In addition, we studied the immune response in the instestine of pigs and reported that immune system plays an extremely important role in the maintenance of gastrointestinal homeostasis. Therefore, we also selected some genes involved with immune responses. Micorarray analysis demonstrated that many immune genes were upregulated in pigs from the ex-evacuation zone in 2012 (Table 2). The gene expression alterations could be considered as evidence of physiological function changes after radiation. Some of the selected genes did not show any changes; however, some showed significant differences. *AIFM1*, *IFN-γ*, and *TLR3* gene expressions were significantly higher in pigs from the ex-evacuation zone than in control pigs (Fig. 1). *Cyclin G1*, *GADD45A*, *XRCC1*, *Smad7*, *XAB2*, *XPC*, *OGG1*, and *EPHX2* gene expressions were similar in both groups. *Cyclin G1* was shown as a representative non-changed gene in Fig. 1. AIFM1 causes mitochondria to release the apoptogenic proteins cytochrome c and caspase-9 [11], INF-γ is one of the important cytokines for host defense and inflammation [12], and TLR3 is associated with radiation resistivity [13] and is involved in antiviral responses. Cyclin G1 is one of the important regulators of the cell cycle. Therefore, changes in the expressions of the genes for these proteins suggested that apoptosis and the immune system in pigs from the ex-evacuation zone might be affected by low-dose radiation.

**Table 2:** The number of significantly differentially expressed genes (>2-fold) linked to immune responses by microarray data analysis

Category	The number of ≥2-fold change genes in category	*p*-Value
Innate immune response in mucosa	2	0.034
Immune effector process	33	0.001
Immune system process	107	0.001
Immune complex clearance	2	0.012
Immune complex clearance by monocytes and macrophages	2	0.012
Regulation of immune system process	51	0.037
Regulation of immune effector process	17	0.010
Positive regulation of immune effector process	10	0.045
Immune response	65	0.001
Positive regulation of immune complex clearance by monocytes and macrophages	2	0.012
Regulation of immune complex clearance by monocytes and macrophages	2	0.012
T-helper 1 type immune response	5	0.007
Type 2 immune response	3	0.050
Innate immune response	35	0.00004
Regulation of innate immune response	15	0.005
Positive regulation of innate immune response	10	0.035

### Gene expression in inobuta

**Fig. 2:**
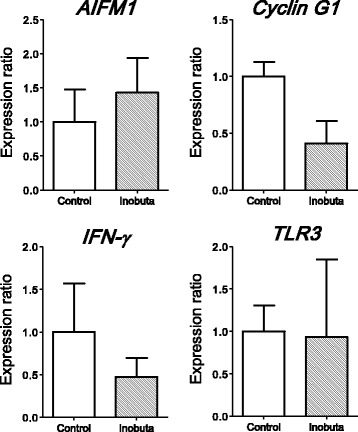
Real-time polymerase chain reaction analysis of *AIFM1*, *Cyclin G1*, *IFN-γ*, and *TLR3* gene expressions in the small intestine of control pigs (*N* = 3) and inobuta from the ex-evacuation zone (*N* = 3). All data are expressed in relative units compared with control pigs. *P < 0.05. Data are presented as mean ± SE

We collected samples from three inobutas at the same time. We analyzed similar gene expressions in the inobutas. Surprisingly, there was no change in gene expressions when compared with the expressions in controls (Fig. 2). However, radioactivity in the longissimus muscle was not high (1000–1400 Bq/kg) in inobuta compared with that in pigs in the ex-evacuation zone.

### Relationship between gene expression and radiation levels in blood

**Fig. 3:**
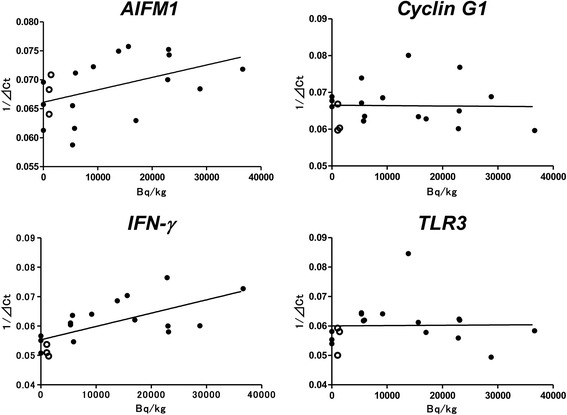
137Cesium radioactivity in skeletal muscles and expression of each gene (black circle: pigs, white circle: inobuta). A positive correlation is observed between muscle radioactivity and expressions of *AIFM1* (R2 = 0.2165) and *IFN-γ* (R2 = 0.4501) but not *Cyclin G1* and *TLR3*

We assessed the relation between gene expression (1/ΔCT) and ^137^Cs radiation. There was a significant correlation with regard to *AIFM1* and *IFN-γ* (Fig. 3). A higher ^137^Cs level in muscles was associated with a higher gene expression level (AIFM1: y = 0.0000002145 ± 0.00000009895x + 0.06613 ± 0.001556 [R^2^ = 0.2165], IFNγ: y = 0.0000004533 ± 0.0000001215x + 0.05532 ± 0.001911 [R^2^ = 0.4501]). There was no correlation between *Cyclin G1* or *TLR3* gene expressions with radiation.

### Gene expressions in the small intestine for samples obtained in 2015

**Fig. 4:**
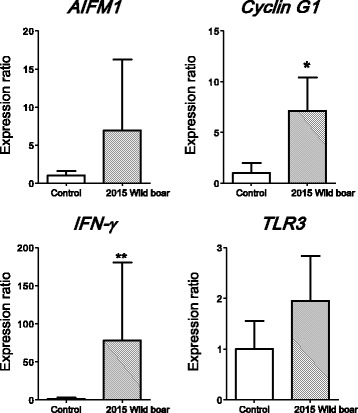
Real-time polymerase chain reaction analysis of *AIFM1*, *Cyclin G1*, *IFN-γ*, and *TLR3* gene expressions in the small intestine of control pigs (*N* = 3) and wild boars from the ex-evacuation zone in 2015 (*N* = 5). All data are expressed in relative units compared with the control pigs. *P < 0.05. Data are presented as mean ± SE

Similar experiments were performed with samples obtained from wild boars in the ex-evacuation zone in 2015. We could not obtain samples from inobuta or pigs at this time point, because they had been eliminated from the area. In addition, we could not obtain a sufficient number of samples, but we investigated whether there was a radiation effect on some genes of interest. Pigs are domesticated wild boars, and both taxonomically belong to the same species (*Sus scrofa*). Therefore, the gene expression of wild boars was analysed using the same primers used for pigs in this study. *Cyclin G1* and *INF-γ* gene expressions were elevated in wild boars in 2015 (Fig. 4). As the number of samples was limited, data variability was large. However, *AIFM1* gene expression also tended to increase in wild boars from the ex-evacuation zone.

**Fig. 5:**
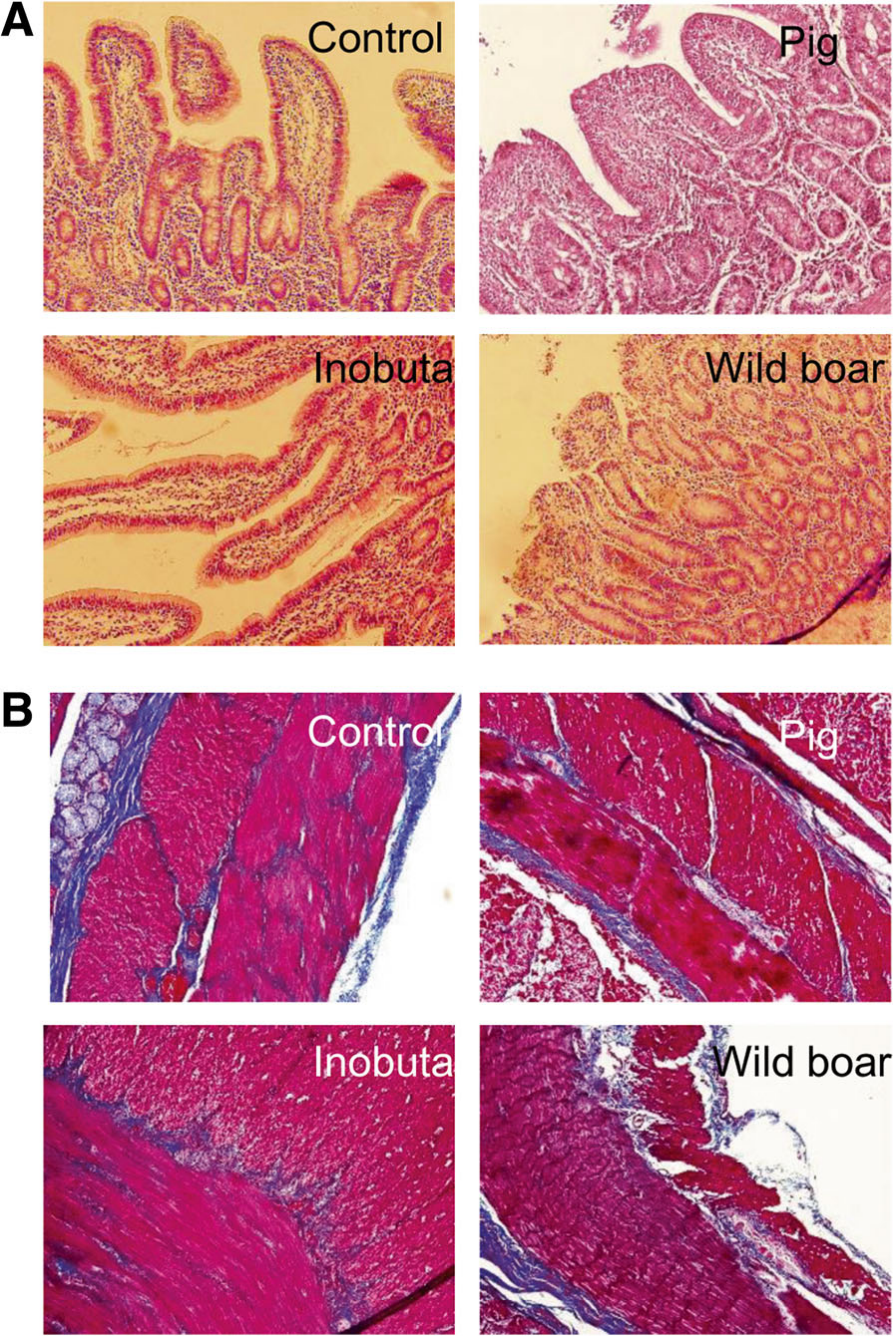
Representative images of hematoxylin and eosin staining (**a**) and Masson trichrome (MT) (**b**) staining of small intestine samples. MT staining shows collagen deposition. However, the results suggest that there were no pathological changes in the small intestine after the accident

To investigate whether intestinal tissues were damaged or showed fibrosis because of radiation exposure, the tissues were fixed and cut for HE staining and MT staining in pathological analysis. MT staining is commonly used to examine collagen deposition in tissue. Despite the highly elevated *AIFM1* and *IFN-γ* gene expressions, there were no morphological changes between the groups (Fig. 5).

## Discussion

There are major concerns about several biological effects of radioactive contamination caused by nuclear power plant accidents. Previous studies on animals and plants in the Fukushima ex-evacuation zone have described biological changes after exposure to radioactive contaminants [14–18]. However, the health consequences of nuclear accidents are still unclear. Therefore, it is important to perform studies in several fields, including developmental biology, immunology, and oncology continuously over a long period. We have been working on host defenses against gastrointestinal nematode parasites in pigs for a long time [6, 19]. As mentioned above, the gastrointestinal tract is an important local immune organ and has extreme sensitivity to radiation, because of the high proliferative activity of epithelial cells. Therefore, whether the intestinal cells in pigs and wild boars are affected after radiation exposure is of interest.

We are members of the ‘Group for Comprehensive Dose Evaluation in Animals from the Area Affected by the Fukushima Nuclear Power Plant Accident’. The sampling method and schedule for sampling, which are discussed in this paper, have been described in previous reports [2, 3, 20, 21]. In this study, we focused on the alterations of gene expressions in the small intestine of pigs in the ex-evacuation zone. Previous research reported that low-dose radiation induced biological responses, such as inflammatory responses, innate immune system activation, and DNA repair [22]. Therefore, we selected several genes associated with inflammation, DNA repair, and the cell cycle for further analysis after microarray analysis. We found that *AIFM1* and *IFN-γ* gene expressions were elevated in pigs from the ex-evacuation zone (Fig. 1). IFN-γ is one of the most important cytokines in type 1 immune responses, and if excess IFN-γ is produced, the intestine shows an inflammatory status. In addition, interferon stimulates several genes, including mediators of apoptosis [23]. In pigs from the ex-evacuation zone, there might be stimulation to eliminate damaged cells in the intestine caused by radiation, inducing apoptosis. Meanwhile, HE and MT staining revealed that there were no morphological changes, including fibrosis, in all tissue samples (Fig. 5). Chronic inflammation in tissue can lead to fibrosis and tissue remodeling; however, the results of this study suggested that the elevation of inflammatory genes was not severe enough to cause morphological changes. Therefore, intestinal homeostasis appeared to be maintained at that time, although inflammatory cytokine and apoptosis-related genes were activated.

The gene expression analysis demonstrated that *TLR3* was also increased. TLRs are very important in the innate immune system. Additionally, they regulate tight junctions in the mucosal barrier system of the intestine. Takemura et al. reported that TLR3 deficient mice showed substantial resistance to gastrointestinal syndrome (GIS) [13]. TLR3 bound to cellular RNA leaking from damaged cells owing to radiation and caused crypt cell death, which can result in GIS. Therefore, the elevation of *TLR3* gene expression might reflect cell response against radiation.

It is important to carefully consider the findings of these gene expressions, because these genes might have been activated by infection. When we obtained samples from the animals, they had no symptoms of infection, such as fever, diarrhea, cough, and wasting. To clarify the relationship between elevated gene expression and radiation, we investigated the relation between the gene expression ratio and the concentration of ^137^Cs in muscles. There were positive relations between *AIFM1* and *IFN-γ* expressions and the concentration of ^137^Cs, but not between *Cyclin G1* and *TLR3* expressions and the concentration of ^137^Cs (Fig. 3). The elevations of *IFN-γ* and *TLR3* in pigs from the ex-evacuation zone might not be regulated by the same mechanism. A previous study by Shea-Donohue et al. investigated the influence of radiation on the cytokine balance in the small intestine and colon in non-human primates and demonstrated an increase in the gene expression of inflammatory cytokines, such as *IFN-γ*, after irradiation [24]. These findings suggest that radiation exposure might result in the alteration of immune genes in animals in the ex-evacuation zone.

Four years after the Great East Japan Earthquake, the air radiation dose has dropped and decontamination of the land has progressed. The government has gradually reduced the ex-evacuation zone. However, there are many wild animals in the evacuation zone. The number of wild boars has increased dramatically, as they breed freely in the ex-evacuation zone. We collected tissue samples from wild boars in the ex-evacuation zone in 2015 to examine gene expressions in the small intestine. *AIFM1*, *Cyclin G1*, *IFN-γ*, and *TLR3* gene expressions tended to be higher in wild boars than in control pigs (Fig. 4). Especially, *Cyclin G1* and *IFN-γ* gene expressions were significantly higher in wild boars than in control pigs. These results suggested that some biological effects might be present in the small intestine after radiation; however, the mechanism is unknown.

## Conclusions

We demonstrated that some changes in gene expression occurred in the small intestine of animals in the ex-evacuation zone after radiation. It is difficult to make a conclusion that these alterations are caused by only artificial radionuclides from the FNPP. However, the animals in the ex-evacuation zone might have experienced some changes due to radioactive materials, including contaminated soil, small animals, and insects. The effects of long-term radiation exposure in living things need to be monitored.
